# Epilepsy in Cambodia–Treatment Aspects and Policy Implications: A Population-Based Representative Survey

**DOI:** 10.1371/journal.pone.0074817

**Published:** 2013-09-05

**Authors:** Devender Bhalla, Kimly Chea, Chamroeun Hun, Vichea Chan, Pierre Huc, Samleng Chan, Robert Sebbag, Daniel Gérard, Michel Dumas, Sophal Oum, Michel Druet-Cabanac, Pierre-Marie Preux

**Affiliations:** 1 Institut National de la Santé et de la Recherche Médicale UMR 1094, Tropical Neuroepidemiology, Limoges, France; 2 Univ. Limoges, School of Medicine, Institute of Neuroepidemiology and Tropical Neurology, Centre national de la recherche scientifique FR 3503 GEIST, Limoges, France; 3 Centre Hospitalier Universitaire, Limoges, France; 4 University of Health Sciences, Phnom Penh, Cambodia; 5 Cambodian Society of Neurology, Phnom Penh, Cambodia; 6 Department of Neurology, Calmette Hospital, Phnom Penh, Cambodia; 7 Department of Access to Medicines, Sanofi, Gentilly, France; University of Ottawa, Canada

## Abstract

**Introduction:**

We tested two treatment strategies to determine: treatment (a) prognosis (seizure frequency, mortality, suicide, and complications), (b) safety and adherence of treatment, (c) self-reported satisfaction with treatment and self-reported productivity, and policy aspects (a) number of required tablets for universal treatment (NRT), (b) cost of management, (c) manpower-gap and requirements for scaling-up of epilepsy care.

**Methods:**

We performed a random-cluster survey (N = 16510) and identified 96 cases (≥1 year of age) in 24 villages. They were screened by using a validated instrument and diagnosed by the neurologists. International guidelines were used for defining and classifying epilepsy. All were given phenobarbital or valproate (cost-free) in two manners patient’s door-steps (March 2009-March 2010, primary-treatment-period, PTP) and treatment through health-centers (March 2010-June 2011, treatment-continuation-period, TCP). The emphasis was to start on a minimum dosage and regime, without any polytherapy, according to the age of the recipients. No titration was done. Seizure-frequency was monthly and self-reported.

**Results:**

The number of seizures reduced from 12.6 (pre-treatment) to 1.2 (end of PTP), following which there was an increase to 3.4 (end of TCP). Between start of PTP and end of TCP, >60.0% became and remained seizure-free. During TCP, ∼26.0% went to health centers to collect their treatment. Complications reduced from 12.5% to 4.2% between start and end of PTP and increased to 17.2% between start and end of TCP. Adverse events reduced from 46.8% to 16.6% between start and end of PTP. Nearly 33 million phenobarbital 100 mg tablets are needed in Cambodia.

**Conclusions:**

Epilepsy responded sufficiently well to the conventional treatment, even when taken at a minimal dosage and a simple daily regimen, without any polytherapy. This is yet another confirmation that it is possible to substantially reduce direct burden of epilepsy through means that are currently available to us.

## Introduction

Epilepsy is a major neurological disorder with particular relevance for low-and-middle-income countries (LMICs). In Asia, people with epilepsy (PWEs) are more likely to die prematurely, remain untreated, and pay more for their treatment cost [1], [2] [3] even when ∼50.0% of the global poverty is present in Asia. Treatment brings necessary reductions in the physical, personal, and social burden consequent to epilepsy [4], yet it remains out of reach for a large number of PWEs [3]. Globally speaking, only few population-based surveys have been conducted on epilepsy in Asia [2] [5], more so on the aspects relating to the treatment [6] [7]. Little local data would mean little possibility of a public health action for the benefit of PWEs. Moreover, many policy aspects such as estimates of the manpower gap, requirements for scaling-up of epilepsy care, the number of anti-epilepsy drug tablets required to be produced, are yet to be defined for many Asian countries. Earlier treatment programs from Asia and the South-East Asia (SEA) [6] [7] have shown major challenges towards achieving a desirable treatment coverage and meeting the needs of PWEs. Cambodia is one of the SEA countries, and in 2009 the lifetime prevalence of epilepsy was 5.8/1000, that translates into an average estimate of nearly 86000 PWEs in Cambodia [5]. Subsequent surveys in Cambodia have shown that 66.0% PWEs remain without optimal treatment or without treatment at all [8], [9]. Many opportunities and challenges with respect to epilepsy have already been identified in Cambodia [8], [9]. The present study builds upon our previous activities in this country [5] [8], [9] while continuing to develop our understanding on epilepsy by bringing newer data from one of the rural populations of SEA. For this study, we aimed to test two different strategies of providing a cost-free conventional treatment to determine: *treatment* (a) prognosis (seizure frequency, mortality, suicide, and complications), (b) treatment safety, (c) adherence to the treatment, (d) self-reported satisfaction with treatment, (e) self-reported productivity, and *policy aspects* (a) number of required tablets for universal treatment, (b) cost of management of epilepsy, (c) manpower gap and requirements for scaling-up of epilepsy care.

## Methods

### Study Location and Selection of Clusters

**Figure 1:**
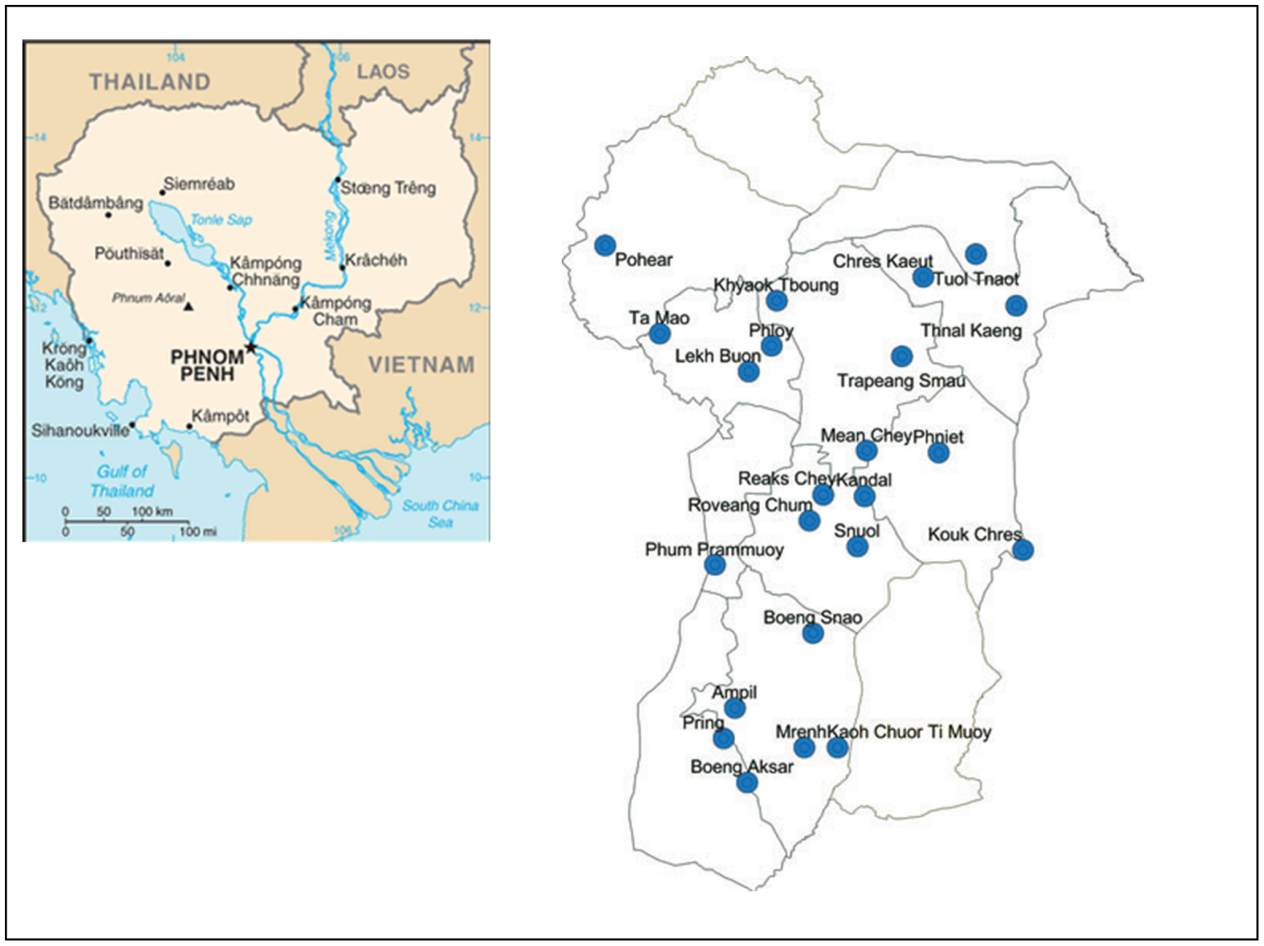
Map and location of Cambodia and the Prey Veng province.

This study was conducted in the Prey Veng province of Cambodia, Figure 1. This province is just about 100 KM from the federal capital, Phnom Penh, which was also the reason for choosing this province for our activities. A random cluster survey design was used in which each village was considered a cluster. These clusters (villages) were randomly selected and so were its constituents that became our study population (as given just below). This province is densely populated (N = 1081609 in 1142 villages) and matches well with the sociodemographic features of Cambodia [10].

### Eligible Study Population and Their Screening for Diagnosing Epilepsy

The study population consisted of all adults and children ≥1 year of age (N = 16510) residing in 24 randomly selected villages of this province. From this study population, all those who had epilepsy (n = 96) were identified by using a door-to-door two-phase procedure: first, screening by using a pre-validated Limoges screening questionnaire for epilepsy in tropical countries, and second, neurological confirmation of the diagnoses by the neurologists [5]. The screening instrument was translated in Khmer and was pre-tested before its final application. All cases also had an electroencephalogram (EEG). Detailed history and examination was conducted for each patient and international guidelines were used for defining and classifying epilepsy [11], [12].

### Follow-up

The study subjects were living in 24 villages, to which access was particularly difficult due to a lack of suitable roads. For treatment and data collection, we followed-up each patient at a pre-defined interval between June 2009 and June 2011. The baseline prevalence survey was conducted in March 2009 subsequent to which the treatment was started. The follow-up (three-monthly) time-points were June 2009, September 2009, December 2009, and June 2011.

Treatment was delivered cost-free (including no user fee) at all times and was provided in two manners. First, at the door-steps (i.e. home-delivery of the treatment supply) of the patients (March 2009 to March 2010, primary-treatment period) in which all patients were given medicines at their home that were sufficient to last for next three months. Second, through primary health centers (PHCs) (March 2010 to June 2011, treatment-continuation period) in which patients were required to go (every three months) to these PHCs and collect their treatment by themselves. Besides free treatment, any subsequent examinations or consultations with the neurologists were kept free-of-cost as well; had these been needed. The only difference in the two treatment periods was that in the primary-treatment period, the treatment was delivered at home of the patients, and in the treatment-continuation period, study participants had to go to PHCs to pick their medications on their own.

All participants were explained the protocol of this study and were informed, at the outset, about the procedures of this study in details, including the need to transition between home-delivery of treatment (primary treatment period) and picking-up treatment from PHCs later-on (treatment continuation period). They were similarly re-called about this transition at the end of the primary treatment period. The staffs at the PHCs in Cambodia are extremely reliable and we worked on the principal of “good-faith”. It seemed unlikely to us that medicines that were provided to the PHCs for the treatment continuation period would instead be sold in the market. If that were the case, it would be difficult to conceal such a step. Local partners were present in the city as well as in the provincial department of health and villages. Patients were also given contact number of collaborators at the provincial health department in case of need or difficulty.

### Treatment Provided

Treatment was with either phenobarbital or valproate for which we used their tablets in the denomination of 100 mg and 500 mg, respectively. The treatment was given by the participating neurologists. While no specific criteria were used to choose one medication over another, we used the best clinical judgment while prescribing a treatment. The emphasis was to start on a minimum recommended dosage and regime, without any polytherapy, according to the body weight of recipients. No titration in particular was done. All treatment decisions (including change in treatment, dosing, etc.) were solely based on the clinical judgement of the treating physician, as is the case in a conventional practice set-up. In addition, each patient received a detailed patient education about epilepsy, possible cause of their epilepsy, and the importance of treatment. All this information was provided in the Khmer language.

### Procedures for Different Outcomes

Treatment aspects are listed below.

#### Prognosis (seizure frequency, mortality, suicide, and complications)

A detailed questionnaire was used to collect data from every participant. Although not validated in Cambodia, this questionnaire has been enormously used in other tropical populations [6]. The seizure frequency for our survey was monthly and was self-reported, along with a confirmation from their suitable proxies. The seizure freedom for this survey was defined as no occurrence of seizure since last visit. Information on mortality (status, circumstances, date, and cause) was determined by conducting verbal autopsy from the immediate family member(s) of the patient. The cause of death was not specifically validated but any relevant documents that a patient’s family might have had were asked to identify the plausible cause of death. Direct method by asking open-ended questions was used to collect data on suicidal thoughts and/or attempts from the patients and was confirmed from their proxies. This was part of the questionnaire and was asked at each visit and to every patient. Suicidal thought was defined as thoughts about suicide or any unusual preoccupation with suicide. This ranged from fleeting to detailed planning, role playing, self-harm and attempts. This was required to have a direct correlation to epilepsy or its treatment. The questionnaire mentioned just above was used to collect data on the epilepsy-related complications and adverse events (AEs) as well. Complications were any physical comorbid event (burning, falls, injuries, etc.) that occurred as a direct result of epilepsy, irrespective of their type. The complications were recorded for a follow-up period and not for a lifetime of the patient.

#### Treatment safety

AEs included any change (physical, mental, behavioral) that had a temporal relation with the treatment intake; all such events were then recorded. We did not use any specific protocol for recording AEs. All patients were specifically asked at each visit to spontaneously report AEs, but without giving them a list of possible AEs to choose from, as is the case in a usual clinical practice.

#### Adherence

Treatment adherence was measured by using pill-count method. The adherence was defined as good in case at least 80.0% of the pills were reported to be consumed by the patients.

#### Patient satisfaction and patient productivity

Self-reported patient’s satisfaction with treatment was determined by asking patients to report on a scale of 0 (worst) to 10 (best) whether they feel better with respect to treatment. Self-reported productivity was determined by asking patients to report on a scale of 0%, 25%, 50.0%, 75.0% and 100%; separately for professional (occupational, earning activities) and non-professional (house jobs, leisure and social) activities. No specific definition of productivity and satisfaction was used. With respect to both satisfaction and productivity, patients were required to analyse their current situation, compare this with their previous state, and then respond how satisfied and productive they themselves feel to be.

#### Treatment continuation

Treatment continuation was determined at the last follow-up visit (June 2011) by requesting each patient to report whether they continued their treatment by going to the PHCs. Treatment continuation was classed according to the duration for which the treatment was taken during its period: <6 months, 6–12 months, >1 year. This doesn’t take into account how frequent patient went to the PHC but the total duration for which the patient went to PHC to collect his/her treatment.

Policy aspects are listed below.

#### Number of required tablets for universal treatment

The required number of tablets was determined for the primary-treatment period alone by taking into account the daily dosage each patient was on and converting this dosage into a number of tablets. For instance someone taking 150 mg of phenobarbital daily would mean a daily consumption of 1.5 tablets of 100 mg phenobarbital. This was done for each patient and for each follow-up point and for overall primary-treatment period, separately for phenobarbital and valproate.

#### Cost of management of epilepsy

The cost of management of epilepsy was determined by using two cost variants: treatment cost and the operational cost. The treatment cost for our patients was determined according to the yearly consumption of tablets (separately for phenobarbital and valproate) by our patients and taking the current prices of 1000 tablets of phenobarbital and valproate (12 and 25 US$, respectively) as a reference. The operational cost included all yearly expenses that were incurred during the management of nearly 100 patients (including materials, transport, per-diems, fieldwork, etc.). This operational cost was internally estimated to be 47,082 US$/year (65.0% of this amount encompassed per-diems for local partners, and rest encompassed transport, materials, travel, and other miscellaneous expenses).

#### Manpower gap and requirements for scaling-up

Unfortunately very little research is done to determine the referential or threshold (i.e. optimal) values that might be used by the countries to determine their needs of the manpower (doctor, neurologists or health worker-doctor, nurse or midwife) and for scaling-up of epilepsy care. For manpower gap, various sources were used to determine the needs of Cambodia with respect to the numbers of neurologists [13], [14], doctors [15] [16], EEGs [15], and health workers (doctor, nurse, or midwife) [17]. For determining the required number of EEGs, a reference value was derived by converting the number of EEGs that are currently known to be available in the African countries into a density (i.e. EEGs per-population; per 100,000). For this a 2005 estimate of the population in Africa was used. With regard to the number of doctors, a reference value was derived from the per-population density of the doctors in each country of the Centre for Integrated Rural Development for Asia and the Pacific (CIRDAP) region and the South Asia’s Association for Regional Cooperation (SAARC) region.

### Ethical Considerations

Ethical clearance was obtained from the National Ethics Committee for Health Research, Ministry of Health of Cambodia, which specifically approved this study. Consent for minors was obtained from the parents, next of kin, or guardians. In rural populations such as in rural Cambodia, written consent is not common as the majority of people are illiterate. Therefore, a form to obtain verbal consent from respondents was proposed and approved, together with the study protocol, by the national Ethics Committee. This was deemed sufficient, as a verbal explanation of the study was necessary anyway. Prior to the interview, our researcher read the consent form carefully in the Khmer language. This consent form contained information on the objectives of the study, the selection process, risks, benefits and freedom of participation, as well as information on confidentiality. Only those who gave their consent were included in the study population; thus, participation was considered to document consent. No potential subject refused to participate.

### Analyses

The main outcome variable was number of seizures. Data entry was carried out by using Epi-info (Epi-info 3.5, Center for disease control and prevention, USA) and data was analyzed using STATA version 9.1 for windows [18]. Seizure numbers were averaged for each time period and a change in this seizure frequency was determined with respect to different time-points and treatment periods. Confidence interval for number of seizures was also determined, and so is the statistical significance (p value) for a change in seizure frequency by using t-test. Complications were estimated for each (and overall) time point, according to the type of complications, and expressed in simple frequencies. A statistical significance of a change from the onset to the end of primary-treatment period, and between periods of primary-treatment and treatment-continuation was determined through chi-squares and respective p values. Mortality and suicide were determined for each time point (and overall period) and expressed in simple numbers. The overall adherence to treatment was analysed for the primary-treatment period alone by averaging the number of those patients who were adjudged to be adherent at each of its time-point. By calculating a chi-square and a respective p value, statistical significance of a change that occurred between onset and the end of the primary-treatment period was determined. Same steps, as with treatment adherence, were performed with regards to the AEs. Self-reported satisfaction with treatment for the overall follow-up period was determined by estimating the number of cases who reported at least 5 (between 0 and 10) as their level of evaluation, at each follow-up point of the primary-treatment period. A group-comparison for determining chi-square and respective p-value was done to analyse any statistical significance of a change between the onset and the end of the primary-treatment period. Same steps were performed, as for self-reported satisfaction with treatment, for analysing data on self-reported productivity. For this, all those who reported to have become efficient (professional activities alone) by at-least 50.0% and at-least 75.0% of their self-determined capacity were taken into consideration. Steps adopted for estimating the required number of tablets, cost of management, manpower gap and scaling-up of epilepsy care are already described in the methods section above. 95% confidence interval (CI) was used and the statistical significance (two-sided) was set at 0.05. For the purpose of data analyses, the study population was considered as «one population group» that is a mix of people of all ages and of all seizure types. This in turn facilitates that our results are closer to a real population and apply to an average set of general population that is a mix of people of all ages and seizure types, rather than to a particular age-group (children or adults) or seizure-type.

## Results

### Clinico-demography

The demographic features, seizure types, and the classification of epilepsy are summarized in table 1 below [5]. In short, our study population had 96 cases and nearly 66.0% were <20 years of age and the most frequent age group was 12–20 years (30.2%) [5]. The average interval period of each follow-up was: 85.5 days (range 79–95) for June 2009, 90.2 days (range 81–93) for September 2009, 94.6 days (range 91–98), and 521.6 days (range 508–538) for June 2011. The total person-days of follow-up were estimated to be 73,911. Nearly 77.0% and 23.0% were treated with phenobarbital and valproate respectively. The frequency of treatments undertaken according to different seizure types were as follows: generalised tonic-clonic (82.0% treated with phenobarbital and 18.0% treated with valproate), absence (0.0% treated with phenobarbital and 100.0% treated with valproate), myoclonic (33.0% treated with phenobarbital and 67.0% treated with valproate), olfactory (100.0% treated with phenobarbital and 0% treated with valproate), partial (75.0% treated with phenobarbital and 25.0% treated with valproate), mixed (83.0% treated with phenobarbital and 17.0% treated with valproate). None of the participant refused to take the treatment. Nearly 41% presented normal EEG. Of the remaining cases, spike and wave discharges occurred in 44%, focal spikes in 21%, generalized slow waves in 19%, and generalized slowing of background in 16%.

**Table 1:** Clinical and demographic features of cases of epilepsy in Prey Veng (Cambodia).

Demographic features		
Number	96	
Males	53.1%	
Median age (years)	24.0 (SD 13.6)	
**Types of seizures**		
*Generalised*	*Partial*	*Others*
GTC (76.0%)	SP, OP and CP (13.5%)	Mixed (6.2%)
Myoclonic (3.1%)		Undefined 0.2%)
Absence (1.0%)		

Classification of epilepsy.

44.8% had idiopathic, 37.4% cryptogenic and 17.8% symptomatic.

Footnotes: GTC: Generalised tonic-clonic; CP: Complex partial: OP: Olfactory partial; SP: Simple partial; SD: Standard deviation.

### Treatment Aspects

Treatment aspects are listed below.

#### Seizure frequency

The number of seizures per month were estimated to be 12.6 (95% CI 8.4–16.7) before the treatment was started. This number reduced sharply with treatment to 1.2 (95% CI 0.4–2.0) seizures per-month at the end of the primary-treatment period, p = 0.00001. After this, the number of seizures increased to 3.4 (95% CI 1.3–5.4) during treatment-continuation period. Globally as a result of primary-treatment, 61.0% stopped having seizures, nearly 23.0% had <3 seizures per-month, and only 16.0% had >3 seizures per-month. The Seizure frequency according to seizure type (non-convulsive: non-generalised tonic-clonic and convulsive: generalised tonic-clonic, respectively) at different time-points of interest were: at baseline (13.20, 95% CI 8.6–17.8 and 12.09, 95% CI 8.3–16.0), at the end of primary-treatment period (1.25, 95% CI 0.44–2.1 and 1.2, 95% CI 0.52–1.9), during treatment-continuation period (3.54, 95% CI 1.24–5.84 and 3.31, 95% CI 1.41–5.21).

#### Epilepsy-related complications

During the overall follow-up period, 10.4% cases on average experienced epilepsy-related complications. The frequency of complications reduced from 12.5% to 4.2% from the start to the end of the primary-treatment period, p = 0.01. During this period, the most frequent complications were fall (67.0%), burns (20.5%), and miscellaneous (12.5%), particularly accident, partial drowning, stroke. Following the end of this primary-treatment period (i.e. during treatment-continuation period), the frequency of complications increased to 17.2% (p = 0.003). Falls (81.2%) were the predominant complication along with burns (18.7%).

#### Mortality

In total, four subjects died during the total follow-up period, one death was related to polio, one to an unknown cause and two were due to drowning during seizures. The two patients who drowned were not on treatment continuation.

#### Suicidal tendency due to epilepsy or its treatment

None of our participant reported that they have had any suicidal thoughts or have attempted one at anytime during the follow-up period.

#### Treatment safety

AEs were several and were highly frequent at the beginning of the primary-treatment period (46.8%, 95% CI 38.8–58.7). This however decreased sharply over time to 16.6% (95% CI 1.0–25.1), p<0.00001 towards the end of this period. The AEs were: s*ystemic:* digestive intolerance (digestive upset, nausea, and diarrhoea) (16.0%), skin reaction with or without fever (21.3%), painful and/or difficult urination (2.0%); *neurotoxic:* fatigue (22.0%), low attention (20.0%), state of confusion (5.2%), lethargy (6.2%), dyskinesia (4.1%), unsteady gait (5.2%), psychomotor slowing (8.1%). For patients who were treated with phenobarbital, the frequency of AEs (during total primary-treatment period) were: s*ystemic:* digestive intolerance (digestive upset, nausea, and diarrhoea) (4.6%), skin reaction with or without fever (9.5%), painful and/or difficult urination (1.0%); *neurotoxic:* fatigue (10.1%), low attention (8.3%), state of confusion (4.2%), lethargy (3.1%), dyskinesia (3.8%), unsteady gait (2.8%), psychomotor slowing (5.9%). For those who were treated with valproate, 16.8% experienced AEs. We give a global frequency only because number of patients treated with VPA was relatively small.

#### Treatment adherence

Patients were observed to be highly adherent (i.e. consumed 80.0% of their tablets) to their treatment during primary-treatment period, the adherence was also observed to increase progressively at each of its follow-up time-points. The difference in the frequency of adherence between start (83.8%) and the end (97.6%) of primary-treatment period was statistically significant (p<0.00001).

#### Self-reported satisfaction with treatment

During the period of primary-treatment, 92.0% cases on average reported to have felt better whilst on treatment. The difference between those who reported to have felt better and those who didn’t was statistically significant (p = 0.00009). This satisfaction progressively increased between start and the end of primary-treatment period from (82.2% to 96.6%, p = 0.001).

#### Self-reported productivity

During the period of primary-treatment, nearly 77.0% patients, on average, were reported to have become productive at at-least 75.0% of their personal capacity, as a result of a one year treatment. The difference between those who became that productive (i.e. at least 75.0% of their personal capacity) and those who didn’t (i.e. <75.0% of their personal capacity) became that productive was statistically significant (p = 0.004). The productivity in life increased progressively between the start and the end of the primary-treatment period (68.7% to 90.0%, p = 0.0003). About 97.0% (exact 96.6%), on average, were reported to have become productive at at-least 50.0% of their personal capacity after one year of treatment. The difference between those who became that productive (i.e. at least 50.0% of their personal capacity) and those who didn’t became that productive (i.e. <50.0% of their personal capacity) was statistically significant (p = 0.002).

#### Treatment continuation

About 26.0% of our patients went to PHCs to collect their treatment at any time during the treatment continuation period. Of these, 30.4% patients had a short-term (<6 months) treatment only.

### Policy Aspects

Policy aspects are listed below.

#### Required number of tablets and their cost

**Figure 2:**
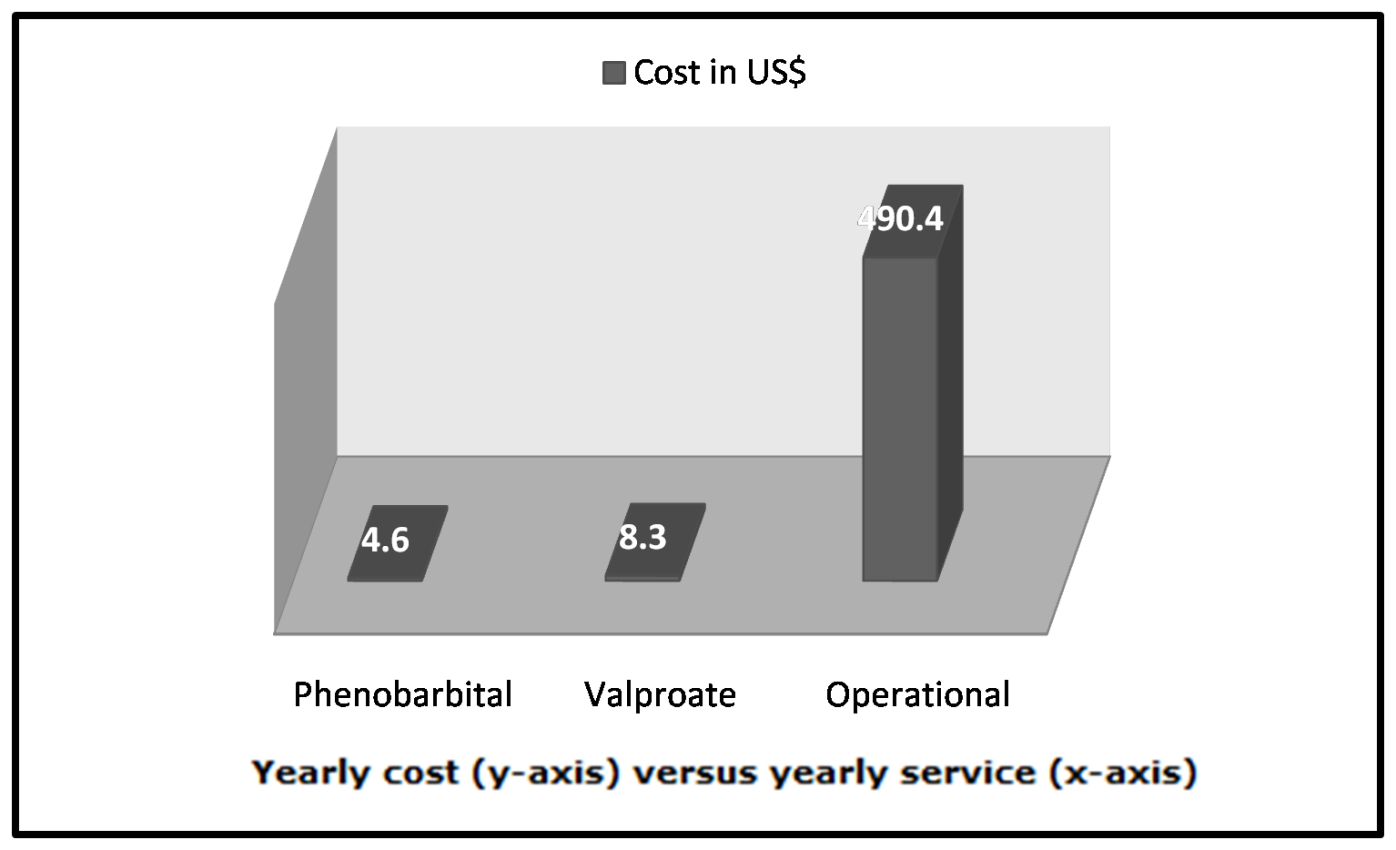
Cost of management (per-patient) of epilepsy through non-local means.

It would require 1.07 (SD 0.29) tablets of phenobarbital (100 mg denomination) daily (and 390.5 tablets yearly) to treat one patient in Cambodia at an annual cost of 4.6 US$. For valproate (500 mg denomination), it would require 0.9 (SD 0.5) daily (333.6 tablets yearly) to treatment one patient in Cambodia at an annual cost of 8.3 US$, figure 2.

#### The cost of conducting a “non-local” case management

Given the total operational cost of 47,082 US$/year (see methods section above) for setting-up a treatment program for 96 patients in rural Cambodia, means that the yearly operational cost for each patient is 490.4 US$. Moreover, the yearly cost of managing one patient is a sum total of per-patient operational cost i.e. 490.4 US$ *plus* per-patient treatment cost (4.6$ for phenobarbital, and 8.3$ for valproate), i.e. 495.0 US$ for managing a patient with phenobarbital and 498.7 US$ for managing a patient with valproate. The management cost is presented in figure 2 below.

#### Manpower-gap and scaling-up of epilepsy care

To overcome the medical manpower gap, Cambodia needs between 148–257 neurologists (0.18–1.0/100,000) [13], 8583–12,874 doctors (0.09–0.6/100,000) [15] [16], and/or 32,901 healthcare workers (doctor, nurse, or midwife) [17].

Besides this, 390.5 tablets of phenobarbital 100 mg per-patient per-year (see relevant section above) would mean that Cambodia requires about 33.5 million tablets of phenobarbital 100 mg a year to provide a 1^st^ year treatment to each of its average 86000 PWEs.

## Discussion

The central aim of conducting epidemiological surveys is to generate a local data that can eventually guide agencies towards taking suitable public health actions. With few data in hand on epilepsy [2] [5] and its treatment [6] [7] in Asia, it is most likely that these countries are unable to take appropriate evidence-based actions. We therefore aimed to conduct a first-ever population-based survey to examine various treatment-related aspects of epilepsy in Cambodia, a SEA country, to guide its agencies towards introducing evidence-based policies. This data not just expands our knowledge on how epilepsy responds to a conventional treatment but also at the same time informs public agencies about the needs and the requirements of epilepsy and people with epilepsy.

A disease state is likely to improve when a suitable and effective treatment is taken. Such is the case with epilepsy for which an effective treatment, such as phenobarbital, is available since 100 years. Globally, it is accepted that 60–70% of epilepsy cases become seizure-free once an optimal treatment is taken [19]. Our results do not differ at all and large proportion of our cases successfully eliminated their seizures after starting their treatment. Our rate of efficacy (seizure elimination) is somewhat better than African surveys [20]–[22] and is just about similar to other Asian surveys [23]. Many factors may play a role in bringing such population-to-population differences, including type or severity of the seizures, comorbid states, dosages used, prior treatment history, the nature, efficacy and the usage of traditional treatments, etc. Some of these factors were observed to clearly influence the treatment efficacy rate in some populations, Mali for instance [24]. Phenobarbital is widely recognised as an effective and practical treatment for LMICs [23]. Although far expensive than phenobarbital, valproate is also an effective and useful treatment of epilepsy in populations that can afford such a treatment [25], [26].

According to one study, only 12.0% epilepsy cases will not experience any side effect from their treatment [27]. In our study, side effects were very frequent at the onset of treatment, but decreased sharply (by 64.6%, p<0.00001) between start and the end of the primary-treatment period. This reduction suggests that the side effects were mild and short lasting, and cleared up spontaneously over a period of few weeks. A high rate of AEs at the beginning of the primary-treatment period suggests that the patients need a habituation period to develop early tolerance to the drugs taken. None of the side effects were such that were unexpected [28]. Although other studies [29] have shown that the occurrence of side effects negatively affect the adherence to the treatment, this wasn’t true for our population. This could be due to the minimal dosages that were used in our population. Similarly, with treatment there was a progressive decline in the rate of complications that the patients experienced due to their epilepsy, such as falls or burns. Between primary-treatment and the treatment-continuation period, the frequency of complications increased considerably, particularly falls. This means that treatment is an important buffer against occurrence of such complications. The predominant presence of complications such as falls and burning could in part be due to the fact that nearly 3/4^th^ of our cases had convulsive seizures.

None of the participant refused to take our treatment for their epilepsy. This implies several important aspects: first is that the patients, family, village and district health authorities had the confidence on the modern treatment of epilepsy and the treatment that was being provided. This also implies that the epilepsy is considered by this population as something that is treatable and by modern therapy. This particular aspect was already shown in another survey of Cambodia where nearly 85.0% of the total study participants had considered epilepsy as a treatable disorder and that by modern therapy [8]. Conversely, elsewhere in Asia, it was noted that PWEs continue to take a traditional therapy even when a cost-free modern treatment is available at the public facilities [30], [31]. Thus, with a positive environment around treatment of epilepsy in Cambodia, it is likely that any large-scale treatment program, if established, would likely to be successful as well. High adherence rate in our population further justifies the acceptability of treatment as well as the presence of a positive environment around epilepsy and its treatment.

Overall 92.0% of our cases reported that they felt better whilst on treatment. This means that the majority of patients were satisfied with the treatment that was given to them. This also means that the response of the treatment was considered adequate and acceptable by the patients, and the treatment given was capable to have met their personal expectations. Epilepsy can manifest in myriad ways and scenarios. It is critical to tease out that irrespective of the varied seizure types and characters, the patient is satisfied that the condition has been corrected (or on its way to get corrected) and that a correct treatment is given to him/her.

Due to epilepsy, PWEs get restricted in their work, education, professional, and day-to-day activities. This restriction affects those with epilepsy more in comparison to those with any other chronic disorder [32], [33]. In the best of their capacity, PWEs can only work half-a-time [34]. In our study, we noted that with treatment nearly 77.0% became productive in their life at at-least 75.0% of their self-determined personal capacity. In Nepal it is seen that about a quarter of cases are generally not able to work up to their personal capacity [31] which matches with our estimation (nearly 3/4^th^ became productive at at-least 75.0% of their personal capacity and 1/4^th^ remained productive at <75.0% of their personal capacity). Furthermore, in our study, the self-reported productivity increased in function of treatment (see relevant section above), providing yet another reason that with treatment it is possible to reduce some of the burden of PWEs. Being active (professionally or otherwise) is essential to maintain a sense of worthiness and self-confidence [35], [36]. With treatment, our cases might to an extent have also improved on their sense of worthiness and self-confidence, although these changes are yet to be measured.

The increased risk of dying prematurely among epilepsy subjects, as compared to the general population, was shown almost a century ago [37]. Despite this increased risk, the absolute number of those who actually die may not necessarily be high [38]. Four cases died mostly as a result of drowning during a seizure. This cause is particularly relevant in our population because working in the rice-fields is the foremost professional activity in this province. Thus those who have epilepsy and work in deep waters of rice fields carry a risk of death by drowning. In other populations as well, drowning during a seizure explains about 5.0% of all deaths due to drowning, although the circumstances in which a death may occur would differ (e.g. shower, lake, pool) [39]. In our study, those who died while drowning were not on the treatment at the pretext of others. Thus, drowning represents a small but preventable cause of death, and being on treatment is likely to prevent such deaths [39]. Another study from a neighboring country (Laos) also showed a high risk of premature mortality as a result of difficult access to the treatment of epilepsy [6].

Suicidal thoughts or attempts are noted with respect to epilepsy or its treatment especially phenobarbital [40]. None of our patient reported to have had suicidal thoughts or attempts as a result of epilepsy or its treatment, at any time-point during the follow-up. The relative absence of such a derogatory practice in our population could be linked to the positive social environment around epilepsy in Cambodia [8]. Presence of positive social environment, such as social support, is likely to render social buffer against such suicidal tendencies (personal data) [8]. Another recent survey has also shown that Cambodians are less likely to be at the risk of suicidal expressions than other populations [41] [42]. Our population is rural that often has close neighborhoods; it would be difficult for someone to hide such suicidal attempts, if any, in such a “visible” environment. Besides this, there is limited stigma in relation to epilepsy in this population [8], [9].

Treatment of epilepsy was provided by using two different strategies: treatment at the door-steps of the patients (primary-treatment period) and treatment through the primary health centers (treatment-continuation period). During both times, the treatment remained cost-free for all patients. Between two periods, a huge increase in the seizure frequency (183.0%) was noted possibly because only a quarter of patients were getting themselves treated during the treatment-continuation period. This implies that the ease of obtaining a treatment is the foremost criterion for the patients to take and remain on treatment, even if the treatment is available at no cost to them. Visibly, the cost-free nature as well as the liberal availability of the treatment did not serve any attraction to most of the patients. Elsewhere, factors such as distance, loss of work, absence of transport, etc. are identified as important barriers to have a treatment (or a regular treatment) [31]. A National Health survey from Cambodia also identified distance to the health facility and no one to accompany as the two foremost barriers of access to a healthcare service [43]. Lack of availability of the treatment is widely recognised as an important barrier towards reaching optimal treatment coverage [3], which our results don’t support. An example of a possible arrangement of the management of epilepsy at the primary-care is presented in table 2.

**Table 2:** Benefits of monthly domestic health visiting (MDHV) and engaging primary health centre (PHC) staff as MDHV.

PHC are local resources (LR) unlike outside resources (OR) (for example a doctor or neurologist from another city) to whom the patient may not be able to relate or confide in completely
Visits by OR may not provide sustained treatment as seizures reduce gradually over a period of time during which using LRs could be advantageous
Engaging PHC staff can facilitate integration of epilepsy into pre-existing set-ups
Dual relevance for pregnancy and birth surveillance as well as for epilepsy
DHV approach matches the need for decentralization of health services in LMICs
Offers a service by providing a *“closer, friendly contact in a home environment”*
Facilitates involvement of other stakeholders (family, village authorities)
Suited to those with special needs (without house, living alone, with comorbid psycho-psychiatric disorders etc.)
Reduced need for visits to a health facility, which are often inadequate due to cost or lack of roads or transport facilities, or long distance or only when complications occur or only when someone is available to accompany the patient
Can help reduce premature treatment cessation through close regular supervision
Can facilitate counselling and monitoring of treatment response, management of side effects or other issues patients may have

We also estimated the optimal manpower needs of Cambodia with respect to the number of general practitioners and the neurologists. By using estimates that were available in the literature, it is clear that the Cambodia is severely lagging behind in the number of general practitioners and the neurologists. A mal-distribution of these resources in Cambodia worsens the situation further. A study from Cambodia shows that most doctors prefer to work in high-payment set-ups, in the capital city, run private clinics and refer hospital patients to their own private clinics [44]. Thus rural populations are getting ignored altogether. There is also a particularly high rate (3.5%) of out-migration of medical doctors from Cambodia [45]. This is crucial for a country that already has a scant manpower. Cambodia currently has 4300 doctors (0.2 doctors per 1000 population) [46] instead of required “ideal” number of 8583–12,874 doctors (0.09–0.6/100,000) [15] [16]. WHO recommends a threshold of 2.3 health workers (doctor, nurse, or midwife) per 1000 population to meet the millennium development goals. Instead of a requirement of 32,901 health workers (doctor, nurse, or midwife), Cambodia currently has one health worker (doctor, nurse, or midwife) per 1000 population in Cambodia (i.e. <50.0% of what is required) [47] . Besides having a sufficient number of healthcare providers, the production and availability of sufficient number of anti-epileptic drugs (AEDs) is also needed. We estimated that Cambodia needs to have about 33 million 100 mg tablets of phenobarbital in order to meet the treatment needs (in the 1^st^ year of treatment) of all of its prevalent cases. Such estimation is rarely conducted although this has obvious significance for policymakers in terms of budget allocation and policy development.

We also saw the «side effects» of the non-local management of epilepsy. We noted that 491 US$ (see relevant section above) were required to be spent yearly for each patient to bring him/her a yearly treatment that cost nearly 1/100^th^ of this amount. Thus, steps should be taken towards making the epilepsy care completely local in nature, table 2. In such an arrangement epilepsy would be expected to be identified locally, treated locally, followed-up locally, and AEDs are procured locally. By engaging non-local agencies to provide epilepsy care, the cost of a patient management raises to an extremely high level. This cost is population-specific and would differ depending upon many factors such as per-diems, travels, etc. Once the epilepsy care becomes completely local in nature, such expenditures can be gainfully avoided, thereby reducing the management cost of PWEs.

A recall bias may have played some role while determining the seizure frequencies which was self-reported in our survey. Despite seeming limitations, this method is still likely to be appropriate for population-based surveys where it is generally not possible to prospectively observe seizures. Moreover, most patients (and their family) have already had a vast experience with their seizures thus it was less likely that they are not aware of what is happening to them and to what extent. Nevertheless, the seizure frequency was also confirmed from the attendants (parents, etc.). Parameters related to cost did not take into account all possible cost sources such as incident cases, cost of loss of productivity, cost of premature death. Similarly, number of required tablets of phenobarbital didn’t take into account the incident cases, remission, or mortality; and this number reflects an estimate for the 1^st^ year of epilepsy treatment alone. We considered our study population as «one population group» that is a mix of people of all ages and of all seizure types. Therefore, no analysis according to the seizure types or the age-groups was intended. Wider confidence intervals of some of our estimates could be due to the small sample size of our study. On a positive note, this is a first-ever attempt to address the parameters, a region, and a country that are only fragmentally addressed. Our data is currently the most suitable to guide health agencies in their aim to devise and implement strategies against epilepsy, both in Cambodia and elsewhere.

## Conclusions

We noted in this population that epilepsy responded sufficiently well to phenobarbital and valproate, even when these were taken at a minimal dosage, without any polytherapy and in a simple daily regimen. These results provide yet another *raison d’être* to the public agencies that it is possible to substantially reduce the burden of epilepsy through means that are currently available to us. Seizures were eliminated in the large proportion of patients, and overall seizure frequency was also substantially reduced. Treatment-related adverse events were considerably frequent at the onset of treatment. These adverse events reduced substantially over time, meaning that these were only transient episodes. Ease of obtaining a treatment is possibly the most major determinant towards reaching desirable treatment coverage as well as to keep patients adherent to their treatment. This is more so for obtaining reductions in seizure frequency, mortality as well as epilepsy-related complications. Cambodia needs to scale-up its epilepsy and general medical services by: having a sufficient number of medical staff, more productive engagement of those human resources that are widely and easily available (e.g. health centre staff), as well as producing sufficient tablets of phenobarbital. For the 1^st^ year of treatment of every patient with epilepsy in Cambodia at this time, about 33 million 100 mg tablets of phenobarbital are needed. This estimate might be used for budget allocation and policy development. There were also strong implied reasons to believe that all patients, family, village, and health authorities had had a high confidence on the modern treatment of epilepsy, and these treatments were acceptable and sufficient to meet the personal expectations of PWEs. None of the participant refused to take a treatment, thus there are less implied reasons to believe that in this population epilepsy is considered as something that has no treatment. There is always a risk for epilepsy-related deaths such as from drowning but many of such deaths are nevertheless preventable. No apparent risk of suicide, either due to epilepsy or its treatment, was noted in this population. Such derogatory practices when reported to be frequent in other populations may unnecessarily generate fear in those populations where there is no apparent risk. Thus it is important that external data is not used for taking health decisions and devising health strategies.

To conclude, epilepsy is an extremely treatable disorder within the means that are currently available to us. Non-use of these available resources in fact creates an ethical dilemma. Progress in global health is not going to be possible without visible progress in epilepsy.
